# Move over endosymbionts, peroxisomes pass electrons too

**DOI:** 10.1042/BST20253101

**Published:** 2025-09-30

**Authors:** Berkley J. Walker, Edward N. Smith, Lee J. Sweetlove

**Affiliations:** 1 Department of Energy Plant Research Laboratory, Michigan State University, East Lansing, MI, 48824, U.S.A.; 2 Department of Plant Biology, Michigan State University, East Lansing, MI, 48824, U.S.A.; 3 Department of Biology, University of Oxford, South Parks Road, Oxford, OX1 3RB, U.K

**Keywords:** fatty acid oxidation, lipids, oxidation reduction, peroxisomes, photorespiration, redox signalling

## Abstract

The importance of the peroxisome as a site of oxidative metabolism in plants is well recognised, but the consequences of peroxisomal biochemistry for the broader metabolic network of plant cells are somewhat overlooked. In this review, we place a spotlight on the peroxisome as a redox-active organelle which mediates substantial flows of electrons. These electron flows not only have consequences within the peroxisome, but they also flow to and from the cytosol and at least two other major redox-active organelles, chloroplasts and mitochondria, with broad implications for metabolism and redox balance of electron carriers such as NADPH and NADH. We will outline the nature of these peroxisome-mediated electron flows and discuss the new appreciation of their quantitative significance derived from metabolic network flux analysis. We emphasise that the flows of reducing equivalents into and out of the peroxisome can be substantial – in some tissues equivalent to that to and from mitochondria. We also highlight key areas of uncertainty around specific redox reactions in the peroxisome and open questions about how redox state is balanced. Finally, we also consider the implications of peroxisomal electron flows in the context of re-engineering key metabolic processes such as photorespiration and lipid accumulation.

## Introduction

The peroxisome is a universal feature of virtually all eukaryotic cells, and oxidative metabolism is a unifying characteristic of the organelle. In plants, peroxisomal oxidative metabolism includes the well-known examples of catabolism of fatty acids by β-oxidation and the oxidation of glycolate produced during photorespiration but also a range of other oxidative reactions. These include β-oxidation steps in the biosynthesis of a number of plant hormones such as indole acetic acid, jasmonic acid and salicylic acid, catabolism of branched-chain amino acids, urate and polyamines and oxidation of sulphite [1]. The final steps of the mevalonic acid pathway are also thought to be localised to peroxisomes [2].

**Figure 1:**
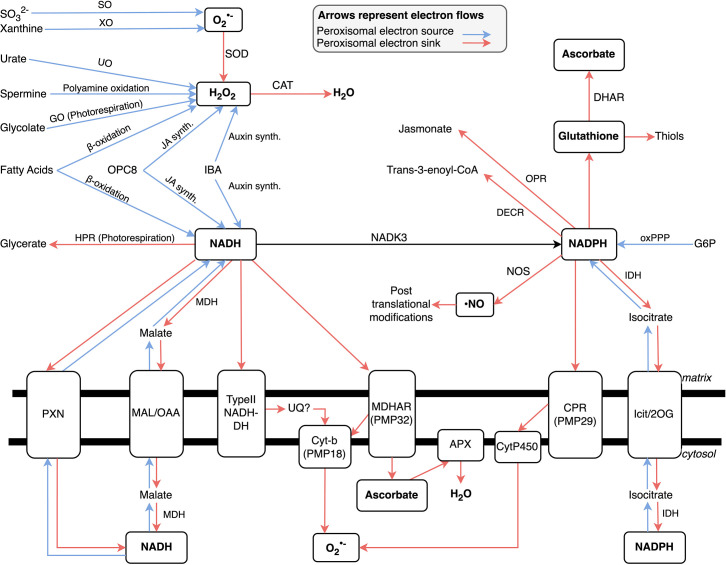
Peroxisomal electron flows. APX, ascorbate peroxidase; CAT, catalase; CPR, cytochrome p450 reductase; DECR, 2,4-dienoyl-CoA reductase; DHAR, dehydroascorbate reductase; GO, glycolate oxidase; HPR, hydroxypyruvate reductase; IBA, Indole-3-butyric acid; IDH, isocitrate dehydrogenase; JA, jasmonic acid; MDH, malate dehydrogenase; MDHAR, monodehydroascorbate reductase; NADK3, NAD kinase; OPC8, 3-oxo-2(2′[Z]-pentenyl)-cyclopentane-1-octanoic acid; OPR, oxophytodienoate-reductase; oxPPP, oxidative pentose phosphate pathway; PXN, peroxisomal nicotinamide adenine dinucleotide carrier; SO, sulphite oxidase; SOD, superoxide dismutase; UQ, ubiquinone; XO, xanthine oxidase.

By definition, oxidation involves the release of electrons. The electrons released by peroxisomal oxidative metabolism flow into various electron carriers, including the peroxisomal NAD^+^ pool, as well as leading to the formation of superoxide and H_2_O_2_ (Figure 1). The redox state of electron carriers may be balanced within the organelle but likely also involves flows of electrons or reducing equivalents between the peroxisome and cytosol and other organelles. During peroxisomal breakdown of fatty acids, for example, electrons are transferred to NAD^+^, leading to a requirement for the resultant NADH to be reoxidised (to regenerate the NAD^+^ electron acceptor). This may be achieved using a peroxisomal membrane-localised electron transport chain or via other metabolic reactions that use NADH as a coenzyme, such as malate dehydrogenase. In leaves in the light, the peroxisomal reactions of photorespiration lead to the peroxisome being a net consumer of NADH, with the reducing equivalents imported from the cytosol likely in the form of malate (Figure 1). Photorespiration also generates large quantities of H_2_O_2_ (Figure 1).

The central role of electron flows within the peroxisome has a deep evolutionary root. The peroxisome is now known to have evolved after the symbiogenic evolution of the mitochondrion, and current thinking is that the evolution of the peroxisome was a direct response to the problem of mitochondrial bioenergetic metabolism [3]. In particular, flows of electrons into the mitochondrial electron chain from the oxidation of different substrates (e.g. carbohydrate versus lipid) generate different ratios of NADH and FADH2, and this can unbalance the redox poise of the chain as the electrons from these two coenzymes enter the chain at different points (complex I and ubiquinone, respectively). The consequence of this imbalance is the detrimental production of reactive oxygen species (ROS) at high rates. It is argued that the evolution of the peroxisome allowed some of that oxidative electron pressure to be shifted out of the mitochondrion, protecting it from ROS-induced damage by allowing flexibility in the NADH and FADH2 ratios. In extant eukaryotes, including plants, peroxisomes are derived from the endoplasmic reticulum, being formed in specialised nest-like structures of endoplasmic reticulum [4], but it is possible that peroxisomes initially evolved from mitochondria via shedding of mitochondrial outer membrane vesicles. Support for this comes from the relatively high proportion of peroxisomal proteins with an ɑ-proteobacterial (i.e. mitochondrial) phylogenetic origin [5] and observations in human fibroblasts that newly formed peroxisomes are derived from a fusion of mitochondria- and ER-derived vesicles [1,6].

This evolutionary context serves to further strengthen the argument that the peroxisome is an important player in electron flows in eukaryotic cells. In the next sections, we discuss quantitative estimates of these electron flows in both autotrophic and heterotrophic plant tissues that strongly argue for a renewed appreciation of peroxisomal metabolism as a major influence on the redox balance and electron balance of the plant cell.

## The peroxisome and photorespiration

While the evolutionary origins of the peroxisome may originally lie in the need to balance redox equivalents and shift oxidative pressures otherwise localised to mitochondria, the peroxisomal function in land plants has since co-evolved in response to specific needs of carbon fixation in an oxygen-rich environment [7]. One main challenge stems from ribulose-1,5-bisphosphate carboxylase/oxygenase (rubisco), which has a moderate affinity for molecular oxygen in addition to its greater specificity for carbon dioxide [8,9]. Reaction between oxygen and ribulose-1,5-bisphosphate produces the inhibitory 2-phosphoglycolate [10–12], which cannot be directly metabolised back into the reductive pentose phosphate (C3) cycle and must therefore be recycled. This recycling is done primarily through photorespiration through the co-ordination of flux through metabolic reactions in the chloroplast, mitochondrion and peroxisome.

During photorespiration, 2-phosphoglycolate is dephosphorylated to glycolate and transported to the peroxisome, where it is oxidised to glyoxylate by glycolate oxidase before receiving an amino group from serine and glutamate or alanine to form glycine. Glycine is transported to the mitochondrion, where it is converted to serine through the co-ordinated activity of the glycine decarboxylase complex and serine hydroxymethyltransferase to produce serine and NADH. Serine is then transported back to the peroxisome, where it provides one half of the amino groups needed to aminate glyoxylate to produce hydroxypyruvate. Hydroxypyruvate is reduced to glycerate by hydroxypyruvate reductase, consuming electrons from NADH. This reaction occurs primarily in the peroxisome, although there is a small amount of cytosolic activity which can occur following genetic perturbation or potentially during periods of excess photorespiratory flux [13,14]. The net electron demand for photorespiration inferred from the above biochemistry is massive in land plants, comprising ~30% of all NAD(P)H reducing equivalents required from autotrophic metabolism [8,15].

While the large net electron demand for photorespiration is assumed to come from reactions outside the peroxisome, there are still major fluxes of electrons in the peroxisome associated with hydroxypyruvate reduction. Redox fluxes of just hydroxypyruvate reductase in the peroxisome comprise a large portion of cellular electron flows. A recent meta-analysis of flux solutions of central metabolism, determined from ^13^CO_2_ labelling, indicates that close to the ~5% of total cellular NAD(P)H produced is consumed in the peroxisome [16]. Similar numbers are determined from a diel flux balance analysis (FBA) of leaf metabolism [17]. When accounting for the net energy demand for photorespiration, plant physiologists have long implicitly assumed that the NADH produced in the mitochondrion from the upstream serine hydroxymethyltransferase offsets the NADH demand for hydroxypyruvate reduction [18–20] but FBA models demonstrate that it is more optimal for the chloroplast to provide the reductant instead and for mitochondrial NADH to be used for ATP synthesis by oxidative phosphorylation [17]. While the exact source of reducing equivalents is important for questions about cellular efficiency and energetics, both hypotheses highlight that the compartmentalisation of photorespiration requires some form of redox shuttling to balance the system (discussed further in ‘Regulation of redox balance in the peroxisome and beyond’).

Regardless of the source of the NADH, hydroxypyruvate reduction in the peroxisome occurs at high rates, and there are additional oxidative reactions that occur with even higher stoichiometric quantities of electrons involved. Specifically, for every two rubisco-catalysed oxygenation reactions, there is one reduction in hydroxypyruvate to glycerate but two glycolate oxidations to glyoxylate. In plants, glycolate oxidation is catalysed by glycolate oxidase, transferring two electrons to molecular oxygen to form H_2_O_2_, but in cyanobacteria, it is catalysed by glycolate dehydrogenase to produce NADH [21]. This raises the question of why plants forego a potential NADH-producing reaction in the peroxisome during photorespiration in lieu of one that produces ROS, especially when NADH is required for a downstream step of the pathway in the same organelle? It is thought the answer to this question is that glycolate dehydrogenase lacks the catalytic efficiency to process the large rates of glycolate production in higher plants, which lack the cyanobacterial carbon concentrating mechanism [22–24]. While likely not the evolutionary driver for glycolate oxidase, H_2_O_2_ signalling resulting from these electron transfer reactions is likely involved in retrograde stress signalling, especially in response to pathogen attack [25,26]. H_2_O_2_ is then detoxified by catalase to form water or by membrane-bound ascorbate peroxidase.

Recently, there has been growing evidence that non-trivial amounts of photorespiratory flux previously assumed to be peroxisomal-localised can take place in the cytosol. These alternative routes include hydroxypyruvate reduction and potentially glyoxylate reduction [13,14] and have recently been reviewed [27]. Cytosolic hydroxypyruvate reduction would decrease electron flow in the peroxisome needed to support photorespiration, utilising reductant in the cytosol instead. Cytosolic glyoxylate reduction would increase peroxisomal electron flow, assuming the resulting glycolate is passed back into the peroxisome for oxidation by glycolate oxidase.

## The peroxisome and lipid catabolism

The peroxisome can also play a dominant metabolic and redox-active role outside of photosynthetic tissues, most notably in the germination of oilseeds, in which high rates of lipid catabolism are used to support growth of the embryonic axis. The process of lipid mobilisation begins with the attack of lipid droplets by lipases releasing free fatty acids, which are taken up by peroxisomes, facilitated by the close physical proximity of the two organelles [28]. Uptake of fatty acids may involve their incorporation into the boundary membrane of the peroxisome and internalisation of lipid vesicles from that membrane [29]. These intralumenal vesicles also contain the enzymes of fatty acid oxidation [29] which involves the oxidation of the β-carbon of the fatty acid (the second carbon following the carboxyl) to a carbonyl, allowing cleavage by a thioesterase enzyme releasing a C2 unit – acetyl CoA [30]. The electrons generated by this oxidation are released as H_2_O_2_ and NADH [31]. Acetyl CoA is further metabolised via the glyoxylate cycle, a variant of the TCA cycle in which the isocitrate dehydrogenase and 2-oxoglutarate dehydrogenase steps are bypassed to avoid losing the two carbons of the fatty-acid-derived acetyl as CO_2_ [32]. Instead, the acetyl carbons are incorporated into C4 carboxylic acids. The glyoxylate cycle enzymes are found both inside and outside of the peroxisome [33]. In the canonical form of the cycle, succinate can be withdrawn, metabolised to malate in mitochondria, converted to oxaloacetate and then phosphoenolpyruvate in the cytosol, facilitating the net conversion of fatty acid carbons into sugars by gluconeogenesis, which are then transferred to the embryonic axis of the germinating seed to fuel its growth.

In oil seeds, where lipids are the main carbon store, the rate of electron generation by peroxisomal β-oxidation of fatty acids is likely to be a dominant flux (and source of electrons) in the storage tissues (typically cotyledons in oilseeds). However, there are no estimates of these fluxes from contemporary fluxomic experiments even though the model plant Arabidopsis and model crops such as *Brassica napus* are oilseeds. This is due to the small size of the seeds and the difficulty of introducing label into the lipid pool. Fortunately, constraint-based metabolic network flux modelling can provide some insight, and one study focusing on soybean seed germination is of particular relevance [34]. Soybean is not an oilseed, with its seeds containing a mixture of storage compounds, typically around 40–45% protein by dry weight [35], 20–35% carbohydrate [36], and about 20% oil [37]. Despite lipid not being the major storage component, the flux modelling suggests that catabolism of lipid stores in the cotyledon 48 hours after germination leads to substantial electron flows, with 24% of total cellular NAD(P)H/NAD(P) metabolism occurring in the peroxisome [34].

## Regulation of redox balance in the peroxisome and beyond

Whether during photorespiration or lipid catabolism, the major destination of the electrons released by oxidative reactions in peroxisomes is the generation of ROS, either directly or indirectly [38]. Oxidase enzymes such as acyl-CoA oxidase and glycolate oxidase generate ROS directly in the form of H_2_O_2_. Peroxisomes contain substantial amounts of catalase to deal with this, with the electrons therefore ending up in water and molecular oxygen. In essence, this can be considered an extended version of the water–water cycle of photosynthesis in the chloroplast [39] since the electrons in the oxidised substrate have ultimately come from splitting of water in photosynthesis. While catalase deals with the majority of H_2_O_2_ within the peroxisome, fine control at lower concentrations of H_2_O_2_ is achieved by ascorbate peroxidase and the associated ascorbate–glutathione cycle with the electrons for the H_2_O_2_ reduction ultimately coming from NADPH [40]. The need to balance detoxification of large amounts of damaging ROS, yet fine control by accessory enzymes at lower concentrations could partially explain why catalase has been selected for; not only is it one of the fastest enzymes characterised [41], but it has low affinity for H_2_O_2_ (e.g. ~1 mM in spinach [42]) relative to ascorbate peroxidases, which have much higher affinity (~20 mM [43,44]).

The electrons for antioxidant metabolism carried by NADPH are ultimately derived from outside the peroxisome in the form of reduced carbon molecules such as glucose 6-phosphate and isocitrate. Glucose-6-phosphate is transported by the glucose 6-phosphate transporter, which is dually localised to the plastid and peroxisome [45]. Glucose-6-phosphate is the initial substrate for the first two steps of the oxidative pentose phosphate pathway catalysed by glucose 6-phosphate dehydrogenase and 6-phosphogluconolactonase, both of which have isoforms found in peroxisomes [46–48]. Isocitrate is the substrate for NADP^+^-dependent peroxisomal isocitrate dehydrogenase [49]. These enzymes transfer electrons from their substrate to NADP^+^, generating NADPH. Other possible routes to balancing the NADPH pool include the enzyme NADH kinase 3, which uses ATP to phosphorylate NADH and the loss of which impairs photorespiratory metabolism [50,51].

In addition to these electron flows via H_2_O_2_, there is also substantial redox activity through the NADH/NAD^+^ pool, with NADH being generated within the peroxisome during β-oxidation reactions. To keep the pool in balance, the NADH must be re-oxidised, and this likely explains the presence of a short electron transport chain within the peroxisomal membrane. The chain is poorly characterised compared with those in chloroplasts and mitochondria but is functionally distinct in that electron fluxes are not coupled to proton motive force generation or ATP synthesis. This is important as it allows requisite flows of electrons required for high oxidation fluxes without limitations imposed by the cellular demand for ATP. One entry point to the chain is a ferricyanide reductase, thought to be the ascorbate–glutathione enzyme, monodehydroascorbate reductase [52] (see later section on ‘open questions in peroxisome redox biology’), that accepts electrons from NADH and transfers them to a cytochrome b [53]. A second entry point for electrons from NADH is via an ‘alternative’ (type II) NADH dehydrogenase of the same type that is also present in chloroplasts and mitochondria [54]. These dehydrogenases pass electrons from NADH (or NADPH) to quinones, and it is likely that the plant peroxisomal membrane contains ubiquinone to accept these electrons [55]. From there, the electrons are presumably passed to cytochrome b, although this has not been experimentally investigated [55]. The terminal electron acceptor of the chain appears to be molecular oxygen, which undergoes single-electron reduction to form the superoxide anion, a potent ROS which would be released to the cytosol [40,56]. The potential ability for peroxisomal reactions to generate, and not just degrade, ROS would cement the peroxisome as a key nexus for plant stress signalling, although the relationship between stress and the production of the superoxide anion from the peroxisome still needs to be resolved more fully [57–59]. Notably, the redox enzymes monodehydroascorbate reductase and malate dehydrogenase, both of which use NADH/NAD^+^, are associated with the peroxisome membrane, and it is often depicted that electrons from these enzymatic processes could also end up in the electron transport chain and generate ROS [60]. Despite these ROS-generating pathways, superoxide anion production from the peroxisomal electron transport chain would only offset a small portion of the total ROS detoxified in the peroxisome.

Another source of redox chemistry in the peroxisome is in the production of the signalling molecule nitric oxide (NO). NO is a radical molecule that mediates various post-translational modifications, including tyrosine nitration (an irreversible process often leading to loss of function) and S-nitrosation (a reversible redox regulatory mechanism that can either inhibit or activate target proteins) [61,62]. There is growing consensus that the signalling role of NO is primarily through these post-translational modifications [63]. These post-translational modifications appear to decrease the activity of several key peroxisomal enzymes involved in redox chemistry, including glycolate oxidase, catalase and malate dehydrogenase [64], with proteomic evidence for additional NO-related post-translation modifications found in the peroxisomal-localised and redox-involved dehydroascorbate reductase, glutathione-disulphide reductase, hydroxypyruvate reductase, isocitrate dehydrogenase and acyl-CoA oxidases [63]. Interestingly, NO is produced in the peroxisome itself via a peroxisomal l-arginine-dependent nitric oxide synthase-like activity [65], raising the hypothesis that ROS and NO signals can interact with each other in the peroxisome [66]. This NO/ROS network is important for cellular adaptation to physiological and environmental changes, including responses to abiotic stresses like salinity, cadmium and lead contamination, where an increase in peroxisomal NO content is associated with a variety of plant responses to stress [59,67–70].

**Figure 2:**
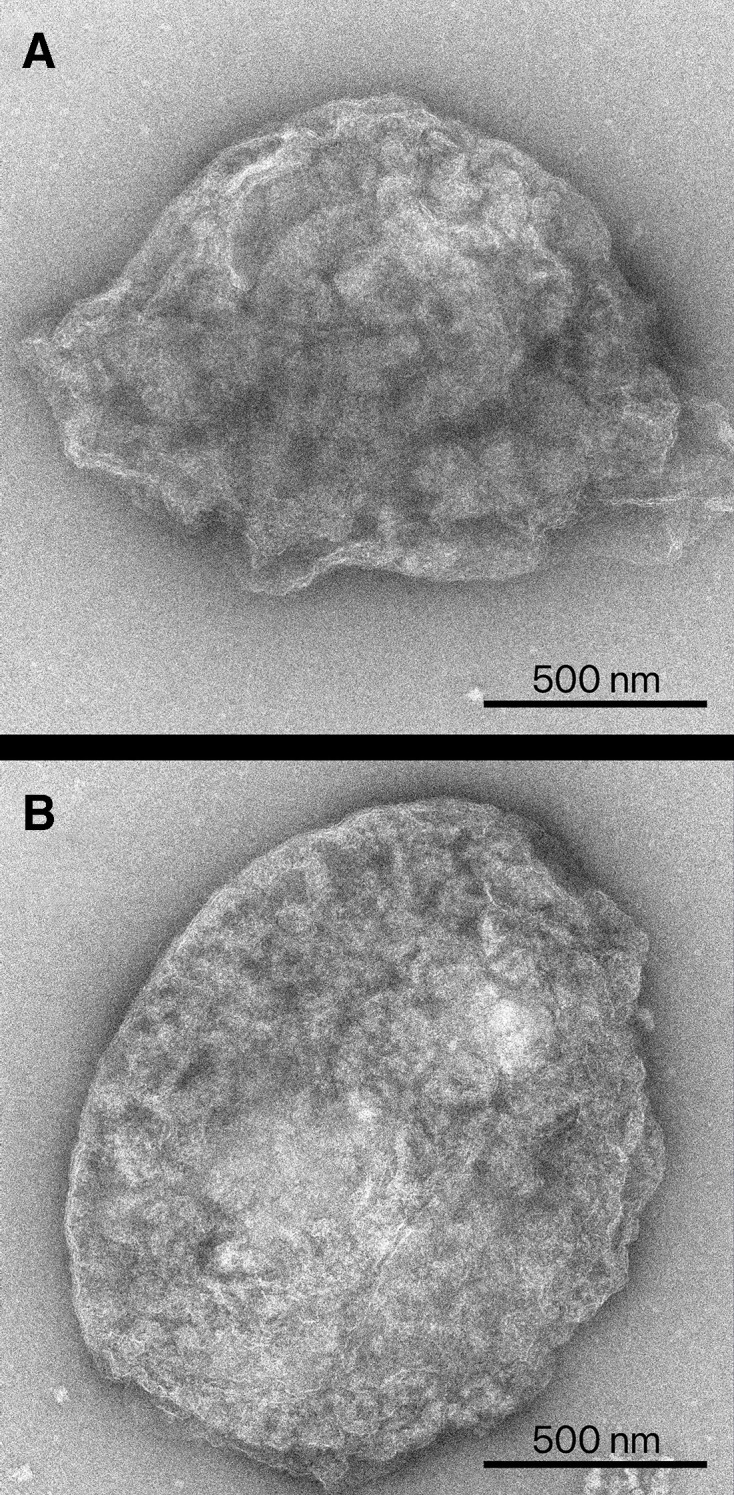
The densely-packed peroxisome. Peroxisomes isolated with an intact membrane (**A**) and with a ruptured membrane (**B**). Isolated organelles were imaged using transmission electron microscopy following negative staining. Note how the main structure of the peroxisome comes from the densely packed protein matrix (**B**), highlighted by the presence of a core that remains intact even without a membrane.

A final important consideration for the regulation of redox balance is the relatively minimal barrier provided by the peroxisomal membrane. The peroxisome appears to contain a structured, electron-dense core in which the key metabolic enzymes are packed together (Figure 2) [71,72]. Physical association between glycolate oxidase and catalase has been demonstrated in leaves and may be a mechanism to minimise or modulate H_2_O_2_ leakage to the cytosol [73]. The peroxisomal membrane, in contrast with the inner envelopes of chloroplasts and mitochondria, is rather freely diffusible to non-bulky anions (such as carboxylic acids but excluding bulkier molecules such as NAD^+^ and ATP) due to the abundant presence of porin-like channels [74]. This free diffusion of metabolites, coupled to the presence of active, near-equilibrium NADH/NAD^+^-utilising enzymes such as malate dehydrogenase in both the peroxisome and cytosol, allows rapid metabolic shuttling of NADH/NAD^+^ (the malate-oxaloacetate shuttle) across the peroxisomal membrane and likely leads to strong cytosol-peroxisome coupling of the NADH/NAD^+^ ratio. The net effect of this is the export of electrons out of the peroxisome (in the form of malate as a reducing equivalent) when excess electrons flow into the peroxisomal NAD^+^ pool. Conversely, when there is a high demand for peroxisomal NADH, as occurs during photorespiration, there will be a net influx of electrons in the form of malate [17]. Hence, the electron flows within the peroxisome directly affect the redox state of the cytosol and likely indirectly affect that of chloroplasts and mitochondria as well [75].

## Open questions in peroxisome redox biology

Considering the central role of peroxisomes involving redox and carbon fluxes, the dense protein matrix of the peroxisomes presents a clear challenge (Figure 2). On the one hand, it must facilitate massive fluxes of intermediates through a small core of proteins. On the other hand, intact peroxisome cores show little activity for most photorespiratory substrates not typically transported between other organelles [71,72]. One explanation for this is tight metabolite channelling or possible metabolon formation, but this has yet to be validated using gold standard techniques like isotope dilution experiments or even protein–protein interaction studies. Additionally, the structure of the protein matrix, or the specific orientation of the enzymes needed to facilitate any metabolite channelling is not clear.

Another open question is how the peroxisomal ascorbate–glutathione cycle is organised. The cycle is a well-established mechanism to reduce hydrogen peroxide to water. The key enzyme is ascorbate peroxidase, with the rest of the cycle functioning to re-reduce ascorbate, the electrons coming from NADPH via glutathione [39]. The enzymes of the cycle are all present inside the peroxisome with the exception of ascorbate peroxidase, which is attached to the boundary membrane (via a single transmembrane pass) with its active N-terminal domain facing the cytosol [76,77]. With ascorbate peroxidase absent from the interior of the peroxisome, how does the internal ascorbate–glutathione function? How does the ascorbate radical, monodehydroascorbate, generated in the cytosol by ascorbate peroxidase, enter the internal cycle? Although the monodehydroascorbate reductase enzyme is also membrane-bound, its active site is thought to face the inside, meaning that monodehydroascorbate must be transported into the peroxisome for further processing [78]. It is unclear whether this would work at appropriate rates to keep the cycle functioning or why such a complex splitting of the cycle between outside and inside is necessary. One suggestion is that the cytosol-facing topology of peroxisomal ascorbate peroxidase could be important to control the amount H_2_O_2_ that is allowed to leak to the cytosol [40]. It is also of interest to resolve the cycle’s functional arrangement under stress, where some aspects of the cycle are increased [79,80].

High-quality proteomics of the peroxisome raises further questions and emphasises the roles of the peroxisome in redox biology [50]. For example, of the 65 proteins identified, 19 were annotated with a redox function. These include major players like hydroxypyruvate reductase and glycolate oxidase, as well as others with roles under autotrophic or heterotrophic metabolism as discussed previously. Also identified was oxophytodienoate reductase, which is involved in jasmonate acid synthesis [81] (Figure 1). Other identified proteins had less clear roles. For example, uricase was found, which breaks down uric acid, a waste product of purine metabolism, into allantoin [82]. While usually thought of in the context of catabolism, allantoin is the primary nitrogen transport molecule of legumes, potentially connecting the peroxisome to whole plant nitrogen metabolism in these species [83,84]. Additionally, sulphite oxidases are found which produce sulphate (Figure 1). In mitochondria, electrons from sulphite oxidase activity are passed to the electron transport chain, but their fate in the peroxisome is not clear. If they do enter the peroxisomal electron transport chain, then they would end up as the superoxide anion (Figure 1) [40].

The role of the peroxisome in developing seeds and stomatal conductance is also not fully resolved. For example, work in developing seeds determined that components of the oxidative pentose phosphate pathway, which growing evidence indicates persists in the light [85–87], may be vital in the peroxisome at least during ovule fertilisation, but the exact role of this is not clear [46]. Additionally, peroxisomal NADPH production is involved in stomatal opening, possibly through signalling stomatal opening [88].

## Engineering peroxisomal electron fluxes using synthetic biology

Synthetic biology strategies can manipulate the electron flows through peroxisomes for biotechnology applications such as production of biomolecules, detoxification or enhanced stress tolerance [89,90]. Photorespiration is responsible for one of the largest electron flows through peroxisomes in illuminated leaves, but alternative pathways have been engineered which are argued to bypass native reactions and divert electron flows from the peroxisome into different organelles. For example, several pathways express glycolate dehydrogenase from *Chlamydomonas reinhardtii* (CrGDH) in the chloroplast in place of glycolate oxidase in the peroxisome [91–94]. This has the net effect of removing both a source of H_2_O_2_ and creating a supply of NADH in the peroxisome. These experiments suggest that the catalytic deficiencies of glycolate dehydrogenase relative to glycolate oxidase may be overcome through transgenic overexpression. Other engineered pathways relocate glycolate oxidase from the peroxisome to the chloroplast and attempt to deal with the additional chloroplast H_2_O_2_ production via chloroplast targeted catalase expression [92,95–98]. The positive effects on yield of these pathways are argued to come from improved energy- and CO_2_-fixation efficiency [99,100] and suggest that peroxisomal electron flows can be safely redirected without detrimental effect to the plant. However, testing plants expressing these engineered pathways in other circumstances where peroxisomal H_2_O_2_ might be important as a signalling molecule or pathogen defence mechanism should be explored to identify if diverting electron fluxes away from peroxisomes can have any negative effect [26,101–103].

Another biotechnological target that involves peroxisomes is as an attractive location for the synthesis of synthetic polymers due to the ready supply of carbon backbones and electrons from the breakdown of fatty acids via β-oxidation. For example, the biodegradable polyesters, polyhydroxyalkanoates, can be synthesised in plant peroxisomes as a method for renewable plastic production [104–108]. Alternatively, native plant lipid synthesis can be engineered for increased production and/or altered lipid profiles [109,110]. However, β-oxidation of lipids in the peroxisome happens at the same time as lipid synthesis [111], and therefore down-regulating β-oxidation, or producing lipids in tissues with low β-oxidation activity such as leaves, can improve the success of engineering efforts [112]. An improved understanding of electron flows required for lipid biosynthesis as well as lipid breakdown in peroxisomes will help improve the success of these efforts.

Another problem that potentially involves engineering peroxisomal metabolism is to deal with biotic and abiotic stresses, such as heat, cold, drought and pathogen infection, which can cause excessive ROS production and cell damage. Engineering ROS metabolism has been explored as a strategy to develop more stress-resilient plants [113,114]. Peroxisome-localised catalase and ascorbate peroxide both catalyse the conversion of H_2_O_2_ to water, and their overexpression or activation has improved salt and osmotic stress tolerance in Arabidopsis [115], peanut (*Arachis hypogaea*) [116] and tobacco (*Nicotiana tabacum*) [117], as well as improving shelf life and cold and drought tolerance in cassava (*Manihot esculenta*) [118,119]. A more general approach to increase ROS detoxifying capacity of plant cells is to increase the number of peroxisomes by over-expressing endogenous peroxisome biogenesis genes [120] or heterologous expression of animal peroxisome proliferator-activated receptors [121]. However, increased peroxisome numbers did not improve salt tolerance in Arabidopsis [122] and led to increased susceptibility to pathogen infection in tobacco [123].

Overall, manipulating peroxisomal electron flow has clear promise for synthetic biology applications [89,90], but the quantitative importance of electron flows through specific redox carriers under specific conditions must be understood for successful application of technologies to the dynamic conditions experienced by crops in the field [114].

**Perspectives**

- Highlights to importance of the field: peroxisomes, long overshadowed by mitochondria and chloroplasts, are now recognised as central hubs of redox metabolism in plant cells. Their substantial electron fluxes influence cellular redox balance, stress signalling and energy distribution, redefining their role in plant physiology and metabolic engineering.
- Summary of current thinking: future research will focus on understanding energy flows through the dense protein core of the peroxisome, elucidating poorly understood redox pathways, and leveraging synthetic biology to rewire peroxisomal metabolism for improved stress resilience, carbon efficiency and biotechnological applications.
- Comment on future directions: the field increasingly views peroxisomes as dynamic redox-active organelles integral to photorespiration, lipid catabolism and reactive oxygen species management. Emerging models emphasise their metabolic connectivity with mitochondria and chloroplasts, highlighting their role in balancing NAD(P)H pools and supporting cellular homeostasis.

## Abbreviations

FBA: flux balance analysis
NO: nitric oxide
ROS: reactive oxygen species
